# Propofol Mediated Protection of the Brain From Ischemia/Reperfusion Injury Through the Regulation of Microglial Connexin 43

**DOI:** 10.3389/fcell.2021.637233

**Published:** 2021-06-08

**Authors:** Tingting Zhang, Yanyan Wang, Qin Xia, Zhiyi Tu, Jiajun Sun, Qi Jing, Pei Chen, Xuan Zhao

**Affiliations:** Department of Anesthesiology, Shanghai Tenth People’s Hospital, Tongji University School of Medicine, Shanghai, China

**Keywords:** Connexin 43, ischemia/reperfusion injury, microglia, propofol, apoptosis

## Abstract

Cerebral ischemia/reperfusion (I/R) injury is a serious condition that leads to increased apoptosis of microglial and neurons in the brain. In this study, we identified that Cx43 expression level is significantly increased in the microglial cells during I/R injury. Using an *in vitro* model (hypoxia/reoxygenation-H/R injury), we observed that H/R injury leads to an increase in activation of microglial cells and increase in levels of pro-inflammatory markers such as IL-1β, IL-6, and TNF-α. Additionally, we could also observe significant increase in phosphorylation of Cx43 and Cav3.2 levels. To assess the role of H/R injured microglial cells on neuronal population, we cultured the neurons with conditioned media (MCS) from H/R injured microglial cells. Interestingly, we observed that microglial H/R injury significantly decreased Map2 expression and affected neuronal morphology. Further, we aimed to assess the effects of propofol on cerebral H/R injury, and observed that 40 μM propofol significantly decreased Cx43, Cx43 phosphorylation, and CaV3.2 levels. Additionally, propofol decreased apoptosis and increased Map2 expression levels in H/R injured neurons. Using silencing experiments, we confirmed that siCx43 could significantly improve the propofol’s rescue after H/R injury in both microglia and neurons. We further developed an *in vivo* MCAO (middle cerebral artery occlusion) rat model to understand the effect of propofol in I/R injury. Interestingly, propofol treatment and downregulation of Cx43 significantly decreased the infract volume and apoptosis in these MCAO rats. Thus, this study clearly establishes that propofol protects the brain against I/R injury through the downregulation of Cx43 in microglial cells.

## Introduction

Ischemia/reperfusion injury (I/R injury) frequently develops during the recovery of blood supply after ischemia in the tissues (Kalogeris et al., 2012). During ischemia, tissues are deficient in oxygen and nutrients from the blood, and when circulation returns, the introduction of oxidative stress contributes to inflammation and oxidative damage (Kurian et al., 2016). Previous studies have shown that sometimes ischemia alone does not cause tissue damage, and that damage usually occurs when blood flow (reperfusion) is abruptly restored after a period of ischemia (Leithead et al., 2013; Jiang et al., 2014; Ponticelli, 2014). Reperfusion to restore blood supply to the brain for treatment of heart and cerebral ischemia might thus leading to brain damage. Situations affecting blood circulation such as surgery, organ transplantation, traumatic shock, thrombosis, and burns could lead to I/R injuries (Collard and Gelman, 2001). However, there are no adequate measures yet in place to prevent/protect organs against I/R injury. Additionally, the incidence of secondary brain I/R injury affecting mortality is high during perioperative time period (Mussa and Taggart, 2002; Ebrahimkhani et al., 2014). Hence, the focus of the anesthetics field has been to identify ways to avoid perioperative I/R cardiac and brain injury.

The major mechanisms involved in I/R injury are excessive production of nitric oxide and other free radicals, inflammation, apoptosis, and homeostasis of calcium (Cheung, 2003). Microglia are immune cells which maintain the brain’s homeostasis in the central nervous system (Taylor and Sansing, 2013). Studies have shown that I/R can activate microglia in the brain and alter its morphology (Yan et al., 2014; Surinkaew et al., 2018). Microglia in turn activates and secretes inflammatory factors (TNFα, IL-1, IL-6, and PGE2) (Cai et al., 2014; He et al., 2016), which are related to neuronal apoptosis (Xia et al., 2015; Zhou et al., 2015). Microglia are neurotransmission regulators, and may affect astrocytes (Pascual et al., 2012). In previous study, a clear significant increase in Iba1^+^ microglia and cell volume increases were observed in cerebral I/R injury brain (Chen et al., 2018). It is also important to understand that I/R affects critical cerebral microcirculation exchange of substances and oxygen supply (Chen et al., 2018).

Connexins are proteins that are widely distributed throughout the body and are critical to the functioning of the heart and brain. Gap junctions created by connexins are required to transmit electrical current between the heart and nerve cells or to coordinate cellular functions through the exchange of chemical signals and energy substrates (Rash et al., 1998; Iyyathurai et al., 2013; Alstrom et al., 2015). Studies have indicated that connexin expression is affected by age and gender (Song et al., 2011; Schulz et al., 2015), as well as pathophysiological changes such as ischemia, post-myocardial remodeling and hypertension (Schulz et al., 2015). Connexin 43 (Cx43) has been identified to be expressed in both the heart and brain tissues and is specifically highly expressed in the astrocytes followed by microglial cells, in the brain tissue (Giaume and Theis, 2010). Inflammatory factors can induce upregulation of Cx43 expression in cerebral capillaries, while inhibition of Cx43 expression may reduce inflammation and promote repair after spinal cord injury (Cronin et al., 2008). Additionally, mimic peptides that inhibit Cx43 activity can minimize vascular leakage and ganglion cell death following retinal ischemia (Danesh-Meyer et al., 2012). The expression of Cx43 is upregulated after CNS injury, and it is thought that Cx43 plays an important role in controlling the degree of this injury. Cx43 plays a major role in I/R injury in the heart and brain and has become a new focus for elucidating I/R injury (Schulz et al., 2015). Propofol and sodium thiopental anesthetics have been known to minimize the exchange of information between gap junctions and weaken the electrophysiological activities in cultivated hippocampal slices (Wentlandt et al., 2006). Propofol can also significantly inhibit the hemichannel activity of LPS-induced cortical astrocytes. Ketamine and dexmedetomidine does also have similar effects, but the inhibitory efficiency is only second to propofol (Liu et al., 2016). Many studies in the recent years have shown that anesthetics (propofol, isoflurane) can inhibit apoptosis, oxidative stress, inflammation and other protective effects on brain I/R (Ji et al., 2015; Tao et al., 2016; Yan et al., 2016).

In summary, cerebral I/R injury affects the activation of microglial cells and induces neuronal apoptosis and additionally it influences the expression and activity of connexins such as Cx43. Further, as anesthetics such as propofol and ketamine have been identified to influence hemichannel activity of gap junctions formed by connexins. Further using *in vitro* and *in vivo* I/R models, we have characterized the I/R damaged microglia, neurons through assessment of Iba1, Cx43 and Cav3.2 levels. Additionally, we also assessed the role of propofol in cerebral I/R injury. Hence, this study clearly establishes the role of Cx43 in the pathogenesis of cerebral I/R injury and identifies the potential mechanism behind propofol’s protection against I/R injury.

## Materials and Methods

### Materials

Propofol (2,6-diisopropylphenol) was obtained from Sigma-Aldrich (United States). For *in vivo* experiments, propofol was used at a dose of 50 mg/kg and for *in vitro* experiments propofol from 5–80 μM concentrations were used. The antibodies used in this study were Anti-CACNA1H (Cav3.2, ab128251, and Abcam), Anti-Connexin 43/GJA1 (phospho S368, ab30559, Abcam), Anti-Connexin 43/GJA1 antibody (ab230537, Abcam), Anti-Iba1 antibody (ab178846, Abcam), Gap27 (HY-P0139, MedChemExpress), and Map2 (ab11268, Abcam).

### Animals

SD male rats (300 ± 10 g) were purchased from Shanghai SLAC Laboratory Animal Co., Ltd. (Shanghai, *n* = 35 in total). Animals were further housed in our animal facility with a 12 h light/dark cycle and 23 ± 1°C temperature (60 ± 10%) humidity. Throughout the study, food and water were provided *ad libitum* for the animals. All experiments were performed with respect to NIH’s principles of laboratory animal care. Further, all the experimental procedures were approved by the Institutional animal care use and ethics committee at the Tongji University School of Medicine.

### *In vivo* I/R MCAO Model

Middle cerebral artery occlusion (MCAO) in SD rats (*n* = 24) were generated using previously published protocol (Longa et al., 1989). Initially, rats were exposed to 5% isoflurane (in N2O/O2, 7:3) to achieve anesthesia and to maintain the rats in anesthetized state 2% isoflurane was employed. The left common and bifurcated carotid artery were exposed through a midline incision in the neck as well as ICA and ECA were separated carefully. Occlusion was achieved by insertion of a silicon rubber-coated monofilament (360 ± 5 μm in diameter) into the external carotid artery and advanced to ICA 18–20mm from the carotid bifurcation. The monofilament was withdrawn after 90 min thus allowing reperfusion. Temperature of the rat was maintained at 37 ± 0.5°C throughout the procedure and lidocaine ointment was applied to the surgical sites to decrease the post-operative pain and supplemented with normal saline when necessary which was similar to the previous study (Lee et al., 2020). Further, validation of the procedure was assessed using neurobehavioral analysis.

### Neurological Evaluation

Neurological evaluation was performed 48 h after induction of ischemia and scores were assigned on a 6-point scale. For no neurological deficit—0, for a failure to extend left forepaw fully- 1, for circling more toward the left- 2, for falling to the left- 3, with no spontaneous movement and with decreased level of consciousness-4, and death-5.

### TTC Staining

Brains were isolated 72 h after I/R and quickly frozen before sectioning into 2 mm coronal sections. Further, the sections were stained using 2% TTC (sigma, United States) in saline solution for 30 min at 37°C. Slices were further imaged and quantified using Image Pro Plus software. Further, the volume of infarction was calculated by multiplying the lesion area and the section thickness.

### Primary Cell Culture

Primary microglial cells were obtained from neonatal SD rats as previously described (Wang and Zhou, 2018). Microglial cells were further cultured in DMEM medium containing 10% FBS and 1% P/S at 37°C in 5% CO_2_ till necessary. Primary rat neuronal cells were obtained from neonatal SD rats as described (Xu et al., 2012). The cells were cultured in neurobasal medium with 10% B27 and 1% P/S.

### H/R *in vitro* Model

H/R injury in the microglial cell model were achieved by culturing the cells in a glucose free culture medium under hypoxic conditions at 37°C with 5% CO_2_ and 1% O_2_ for 2–24 h. Further, reoxygenation of cells were performed with 5% CO_2_ and 21% O_2_ for 12 h in full culture medium. Based on the results from MTT assay, we choose 12 h as the hypoxia time point. All subsequent experiments were carried out with 12 h of hypoxia and 12 h of reoxygenation (H_12_/R_12_). Microglial cells underwent H/R injury and after 24 h (H12/R12), microglial cell supernatant (MCS) were collected and centrifuged at 1,500 rpm for 5 min. Further, neuronal cells were cultured with the MCS medium for 24 h to assess the role of H/R injury onto the neuronal cells.

### MTT Assay

On 96 well plate, cells were initially seeded. After H/R injury, wells were cultured with Methylthiazolyldiphenyl-tetrazolium bromide (MTT) (0.5 mg/ml, Sigma) in a humidified incubator at 37°C (5% CO2) for 90 min. Further, cells were treated with 100 μl/well of DMSO for 30 min in dark. The supernatant was transferred to a flat-bottom 96-well microtiter plate and measured at 560 nm using a spectrometer.

### ELISA Assay

To assess IL-1β, IL-6, TNF-α, and IL-10 levels, we used ELISA assays obtained from Shanghai Jianglai Bio., and was performed according to the manufacturer’s instructions. The absorbance was detected using a plate reader at the absorption of 405 nm.

### Immunofluorescence

The cells or were fixed using 4% paraformaldehyde at room temperature (RT) for 15 min. The cells were then washed thoroughly with PBS and permeabilized with 0.25% triton-x in PBS for 10 min. Cells were blocked with 1% BSA in PBST (PBS with 0.1% Tween-20) for 30 min. Further, the cells were incubated with CACNA1H, Connexin 43, Map2 and Iba1 antibody primary antibodies overnight at 4°C. The cells were thoroughly washed with PBST and incubated with corresponding secondary antibodies for 1 h at RT. Again the cells were washed with PBST and stained with DAPI for 5 min. Finally, the cells were visualized and imaged using a fluorescence microscope (Leica, Germany). Immunofluorescence staining was also performed in the hippocampus and its adjacent tissues.

### TUNEL

Cells were seeded onto dishes and allowed to attach for 12 h and undergo H/R, the TUNEL staining was carried out according to the manufacturer’s instructions (Roche). Finally, the cells were visualized and imaged using a fluorescence microscope (Leica, Germany).

### Western Blotting

Protein extraction was carried out using whole protein extraction kit (Solarbio, China). Further, protein concentration was assessed using Bradford assay. A total 20 μg of the protein were loaded onto SDS-PAGE gel (4–15%) and allowed to migrate for 1 h. Further, the gel was transferred onto a nitrocellulose membrane and blocked using 5% skim milk for 1 h. Next, the membrane was incubated with respective primary antibodies overnight at 4°C. The membranes were thoroughly washed with PBS-T and then incubated with corresponding secondary antibodies for 1 h at RT. The membranes were again washed and then incubated with chemiluminescence substrate (Thermo Scientific^TM^) for 10 min followed by being imaged using a reader 60.

### Statistical Analysis

Data were assessed with Student’s *t*-test and with One way Analysis of Variance (ANOVA) in three and more groups using GraphPad Prism. All the data is presented as mean ± the standard deviation (SD). Statistical significance is indicated on the figures as ^∗^*p* < 0.05, ^∗∗^*p* < 0.01.

## Results

### H/R Induces Microglia Activation With Increasing Cx43 Phosphorylation and Cav3.2 Expression

**FIGURE 1:**
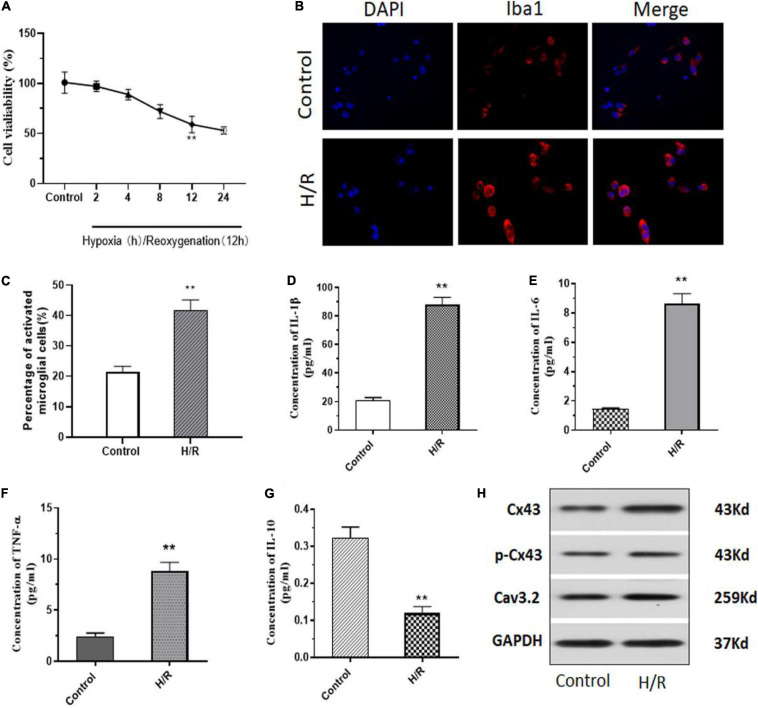
H/R induces microglia activation with an increase in Cx43 phosphorylation and Cav3.2 expression. **(A)** Cell viability as measured using MTT assay. **(B)** H/R induced activation of microglia with increasing Iba1 expression. **(C)** Number of Iba1^+^ cells (activated microglia) as assessed using ImageJ. **(D–G)** The levels of inflammatory cytokines of IL-1β, IL-6, TNF-α, and IL-10 were analyzed using ELISA. **(H)** Protein expression of Cx43, phosphorylation Cx43 and Cav3.2 as observed through western blotting. *p < 0.05, ** *p* < 0.01 *vs.* Control. *n* = 3.

Initially, to assess the role of H/R injury on cells, we isolated primary microglial cells from neonatal SD rats and induced H/R injury. All cells were treated in hypoxic conditions for 2–24 h, further the cells were reoxygenated for 12 h. Evidentially, we observed a significant decrease in cell viability after 8–12 h of hypoxia which was further sustained till 24 h of hypoxia (Figure 1A). For subsequent experiments, all cells were treated with 12 h of hypoxia followed by 12 h of reoxygenation. Next, we performed some immunofluorescence (IF) staining on the cells after H/R injury and observed a significant 20% increase in Iba1^+^ cells, indicating an increase in microglial activation after H/R injury (Figures 1B,C). Additionally, we analyzed the levels of inflammatory cytokines such as interleukin-1β (IL-1β), IL-6, tumor necrosis factor (TNF-α) and IL-10 after HR injury. We observed a significant increase in IL-1β, IL-6, and TNF-α (Figures 1D–F). However, we observed a significant decrease in the levels of IL-10 (Figure 1G). Additionally, using western blotting we observed that post H/R, cells expressed increased levels of Cx43 and phosphorylated Cx43 (p-Cx43) (Figure 1H). Interestingly, we also observed a significant increase in the expression levels of T-type voltage gated calcium channel, Cav3.2, after exposure to H/R (Figure 1H).

### H/R Induces Microglial Apoptosis and Neuronal Morphological Impairment Through Microglia Activation

**FIGURE 2:**
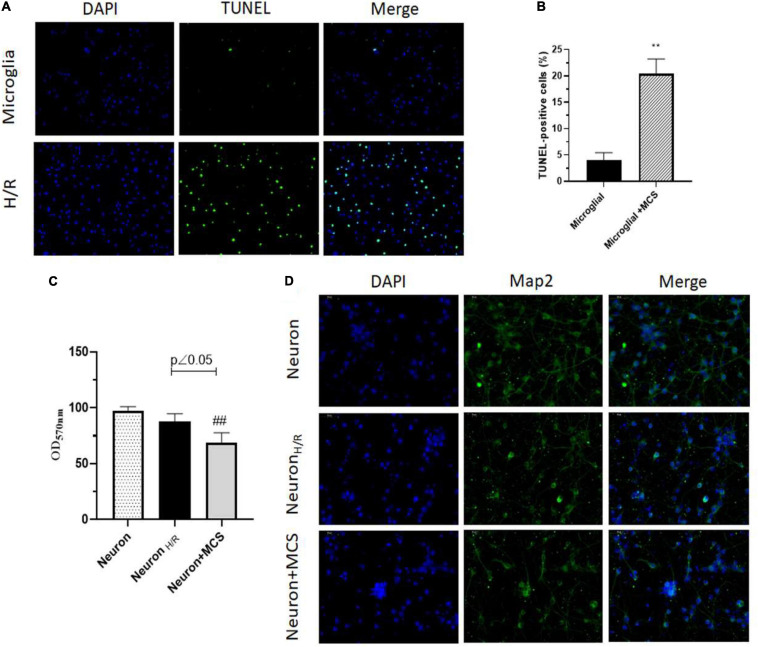
H/R induces microglial apoptosis and neuronal morphological impairment through microglia activation. **(A)** TUNEL assay of microglia (Cultured under normal conditions), H/R induced microglia (H/R). **(B)** TUNEL-positive cells were analyzed using ImageJ. **(C)** Cell viability assayed by MTT. **(D)** Map2 expression of neuron detected using immunofluorescence staining. ^∗∗^*p* < 0.01 *vs.* Microglia. ##*p* < 0.01 *vs*. Neuron _H/R_. *n* = 3.

To identify the role of H/R injury on cell apoptosis, we performed TUNEL assay. And, we observed that H/R significantly increased apoptosis in microglial cells (Figures 2A,B). Further, to understand the role of H/R injury in neurons, we used the supernatant of H/R injured microglial cells (MCS, microglial cell supernatant) and cultured the rat primary neurons for 24 h. Interestingly, using MTT assay, we observed that the viability of neurons decreased slightly after H/R injury, but the viability decreased more significantly after culture with MCS (Figure 2C). Further, using immunofluorescence staining, we observed a clear decrease in MAP2 positive neurons along with a decrease in axonal structures post H/R injury and treatment with MCS (Figure 2D). These results indicated the negative deleterious effects of H/R injured microglial cells on neural viability and morphology.

### Propofol Inhibited the H/R Induced Activation and Cx43 Expression in Microglia

**FIGURE 3:**
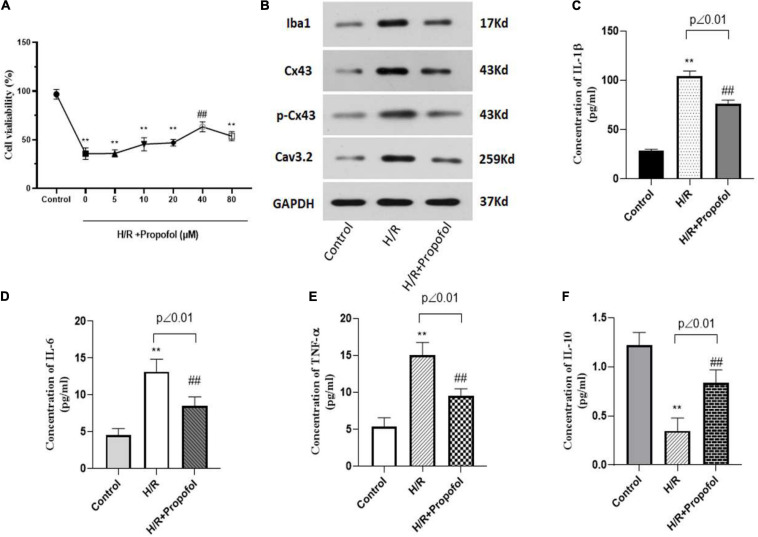
Propofol inhibited the H/R induced activation and Cx43 expression in microglia. **(A)** Cell viability of H/R induced microglia treated with 0–80 μM of propofol as observed using MTT assay. For the following experiments, we used 40 μM of propofol for the treatment of microglia. **(B)** Protein expression of Iba1, Cx43, phosphorylated Cx43 and Cav3.2 was measured using western blotting. **(C–F)** The levels of inflammatory cytokines of IL-1β, IL-6, TNF-α, and IL-10 were measured by ELISA kit. **p < 0.01 *vs*. control, ##*p* < 0.01 *vs*. H/R. *n* = 3.

As previous studies have indicated a potential role for propofol on I/R injury (Tao et al., 2016), in our study, we aimed to explore the role of propofol in microglial activation mediated neuron apoptosis and Cx43 expression involved in. To achieve this, initially primary rat microglial cells were H/R injured and then treated with propofol at varying concentrations from 0–80 μM for 24 h. Using MTT assay, we observed that post treatment with propofol there was a slight increase in H/R injured cell’s viability. However, with 40 μM propofol treatment there was a significant increase in cell viability, when compared to the untreated H/R injured microglial cells (Figure 3A). Further, we also observed that 40 μM propofol could significantly decrease Iba1, Cx43, Cx43 phosphorylation and Cav3.2 expression levels (Figure 3B). These results indicated that propofol could clearly decrease microglial activation and decrease phosphorylation and activation of Cx43. Additionally, we also observed a significant decrease in inflammatory cytokines such as IL-1β, IL-6, and TNF-α (Figures 3C–E). However, we also observed a significant increase in the levels of IL-10 levels (Figure 3F). Hence, propofol treatment could significantly rescue the microglial cells from the deleterious effects of H/R injury. Our results demonstrated that protects the brain against I/R injury through the downregulation of Cx43 in microglial cells to decreased neuron apoptosis.

### Knock Down of Cx43 Enhances the Effect of Propofol on Microglia Mediated Neuron Impairment Induced by H/R

**FIGURE 4:**
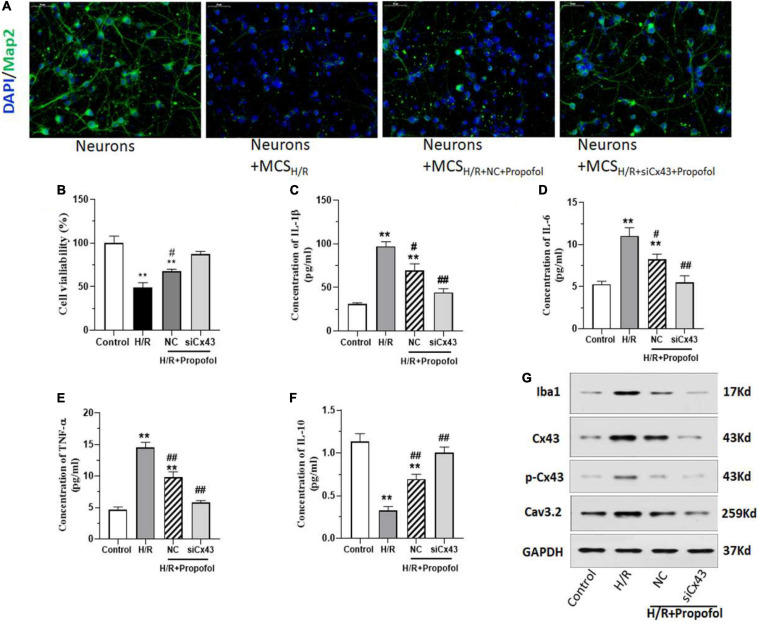
Silencing of Cx43 expression enhances the effect of propofol on microglia mediated neuron impairment induced by H/R. **(A)** Expression of Map2 in neurons after treatment with/without microglial cell supernatant after H/R (MCS), treated with/without propofol and transfected with negative control (NC) or siRNA for Cx43 (siCx43) was measured using immunofluorescence staining. **(B)** MTT cell viability assay of microglial cells which were w/wo H/R injury, transfected either with NC or siCx43 and treated with propofol. **(C–F)** The inflammatory cytokine IL-1β, IL-6, TNF-α, and IL-10 levels were measured using ELISA. **(G)** The protein expression of Iba1, Cx43, phosphorylation Cx43 (p-Cx43) and Cav3.2 was detected by western blotting. *p < 0.05, **p < 0.01 *vs*. Control. #*p* < 0.05, ##*p* < 0.01 *vs*. H/R. *n* = 3.

Based on the above-mentioned results, it was clear that activation of Cx43 plays a vital role in H/R injury. Hence, we wanted to understand further if lack of Cx43 could affect propofol rescue on H/R induced neuronal impairment. Similar to previous results propofol could rescue the Map2 expression and neuronal morphology in the cells treated with H/R exposed MCS (Figure 4A). However, when the cells were silenced for Cx43 expression, there was a significantly higher rescue of Map2 expression and a higher morphological recovery among neurons (Figure 4A). Further, silencing of Cx43 and propofol treatment significantly rescued the cell viability of microglial cells, when compared to the H/R injured cells (Figure 4B). Additionally, silencing of Cx43 along with propofol treatment significantly decreased the inflammatory marker levels (IL-1β, IL-6, and TNF-α) (Figures 4C–E). However, we also observed a significant increase in the levels of IL-10 levels (Figure 4F). Further, we assessed the levels of Iba1, Cx43, Cx43 phosphorylation and Cav3.2 levels using western blotting and based on these results it was clear that, silencing of Cx43 and propofol treatment could initially significantly decrease the expression of levels Cx43 and Cx43 phosphorylation (Figure 4G). Additionally, it could also significantly decrease Iba1 levels, thereby indicating a decreased activation of microglial cells (Figure 4G). Similarly, we also observed a significant decrease in Cav3.2 levels. Interestingly, all these above-mentioned markers displayed levels almost similarly to the control uninjured cells. This indicated a potential complete rescue of the deleterious effects of H/R injury with the aid of Cx43 silencing and propofol treatment.

### Propofol Alleviates H/R-Induced Neurological Impairment by Decreasing Cx43 and Cav3.2

**FIGURE 5:**
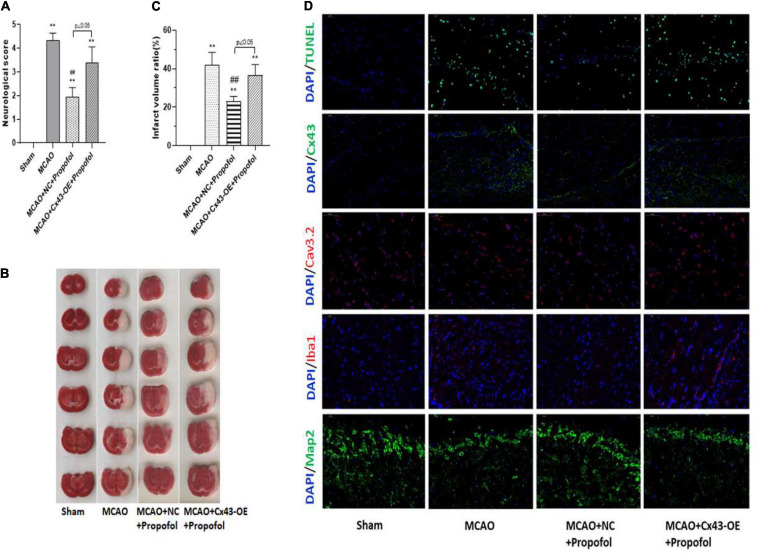
Propofol alleviates H/R-induced neurological impairment by decreasing Cx43 and Cav3.2. Rat middle cerebral artery occlusion (MCAO) model was generated and animals were treated with/without propofol and intraventricularly injected with negative control (NC) or Cx43 overexpression lentivirus (Cx 43-OE). Sham group is used as the control here. **(A)** Neurobehavioral analysis is performed and scores are assessed. **(B)** TTC staining is used to assess the cerebral infarction, and the infarct volume was analyzed. **(C)** Apoptosis was measured using TUNEL staining and the protein expression and localization of Cx43, Cav3.2, Iba1, and Map2 was analyzed by immunofluorescence staining. *p < 0.05, **p < 0.01 *vs.* Sham. #*p* < 0.05, ##*p* < 0.01 *vs*. MCAO. *n* = 3.

Further, we generated a rat middle cerebral artery occlusion (MCAO) model and checked the effect of propofol in rescuing induced injury. To confirm that propofol alleviates the H/R induced injury through the regulation of Cx43, we overexpressed Cx43 in the MCAO models. The rats were first separated into four groups: sham (untreated rats), MCAO (injured rats), MCAO treated with propofol and injected with negative control (empty plasmid) for Cx43 (MCAO + NC + propofol) and MCAO treated with propofol and intravenously injected with overexpression lentivirus for Cx43 (MCAO + Cx43-OE + propofol). We first assessed the neurobehavioral analysis score of these rats and observed that MCAO rats had a high neurological score, whereas treatment with propofol significantly decreased this score (Figure 5A). However, overexpression with Cx43 increased and worsened the behavior of these rats. Further, we visualized and measured the cerebral infract volume using TTC staining and confirmed that the MCAO rats had a significantly higher injury/infract volume (Figure 5B). Further, we observed that the propofol treatment significantly decreased the injury or infract volume in these MCAO rats. However, Cx43 overexpression significantly decreased this recovery achieved by propofol. Further, we assessed vital markers such as TUNEL, Cx43, Cav3.2, Iba1, Map2 (Figure 5C). Initially, Cx43 staining confirmed that MCAO rats displayed high levels of Cx43 and propofol treatment clearly decreased Cx43 levels. Further, however, propofol treatment could not efficiently decrease the Cx43 levels in the Cx43 overexpression group. Further, TUNEL staining showed that the MCAO rats displayed increased levels of apoptosis, which could be evidentially decreased by propofol treatment. However, Cx43 overexpression, further increased the apoptosis levels despite the propofol treatment. Additionally, we could also observe an increase in number of cells expressing Iba1 and Cav3.2 in the MCAO rats compared to the sham rats. Propofol treatment could evidently decrease Iba1 and Cav3.2 positive cells in the MCAO rats. However, Cx43 overexpression increased the levels of Iba1 and Cav3.2. Similar to our *in vitro* results, MCAO models also displayed decreased Map2 positive neuronal cells, however, propofol treatment could significantly recover this decrease. But, Cx43 overexpression did clearly worsen the Map2 expression levels even in the presence of propofol treatment. However, it is noteworthy that with all the above-mentioned evidence, it was clear that the even in the presence of Cx43 overexpression, propofol treatment could still decrease Cx43 levels and slightly recover the H/R associated injury. These results clearly indicate that propofol alleviates H/R induced damage by decreasing Cx43 and Cav3.2 levels.

## Discussion

I/R associated injury to the organs remain as the most prevalent inflammation associated damage to tissues and cells. In this study, we aimed to assess its role in brain microglial and we identified that H/R injury induces the activation of the microglial cells. Further, it was clear that H/R injury increases microglial cell death, and release of pro-inflammatory markers such as IL-1β, IL-6, TNF-α. Additionally, we observed an increased expression of Iba1 after exposure to H/R. Iba1 is a prevalently used marker indicating the macrophage activation levels and clearly our results illustrated the status of the macrophages after H/R injury (Davis et al., 2017). Interestingly, we also observed that it significantly increased the levels of Cx43 (Figure 1). In the human system, glial cells encompasses 91% of cell populations in the central nervous system (Allen and Barres, 2009). Interactions between glia and neuronal cells are necessary for transmission of key information survival and functioning of the two populations (Giaume and Theis, 2010). This intercellular interaction is achieved primarily through gap junctions or through hemichannels. One such protein family are the connexins (cxs). Cxs as gap junctions allow cell-cell interactions and exchanges of ions and small molecules, whereas as hemichannels they allow release of factors and other molecules into the external medium (Harris, 2007). Cx43 is majorly expressed in glial cells and vascular endothelial cells, and are identified to be primarily expressed after spinal injury (Naus et al., 1991; Danesh-Meyer et al., 2012). Importantly, it has been observed that upregulation of Cx43 immediately after ischemia may increase spreading of apoptotic signals, cellular debris and other dying cells to neighboring healthy environments thereby compounding the injury to the tissue and initiating new cell death signals (Frantseva et al., 2002). Cx43 upregulation has been observed in many ischemic diseases such as stroke, brain ischemia, brain and spinal cord injury (Hossain et al., 1994; Nakase et al., 2004; Ohsumi et al., 2006). Many recent studies have focused on the decrease of such gap junctions using small molecules or peptides post injury (Danesh-Meyer et al., 2008). However, it is essential to identify strategies which is employable either during pre-operative or peri-operative situations to minimize surgical associated I/R injury. Additionally, another key observation form our results are increased expression of CaV3.2 calcium channels after H/R injury, which has been associated with increased Ca^2+^ ion entry leading to mitochondrial overload and thus increased cell death under ischemic conditions (Gouriou et al., 2013).

Hence, from our evidence it was clear that microglial cells were highly affected by H/R injury. However, we further wanted to assess the effect of H/R injured microglial cells on neuronal cells. Interestingly, we observed that the MCS from H/R injured cells significantly decreased Map2 expression and increased apoptosis of neuronal cells (Figure 2). These results clearly indicated the effect of H/R injury on neuronal cells through the release of factors by H/R injured microglia cells. The effect of Cx43 hemichannels releasing microglial pro-inflammatory along with other paracrine and autocrine factors could significantly affect the morphology and physiological state of neuronal cells. These results clearly indicated for a need to develop treatment strategies associated with downregulation of hemichannel and gap junction associated proteins during I/R injury. Previously, studies have indicated the effect of anesthetics to inhibit gap junction and hemichannel activity in both astrocytes and neurons (Liu et al., 2016). Initially, we identified that propofol indeed does increase the cell viability and decrease Cx43 expression levels in H/R injured cell models. Additionally, we observed that propofol decreases Iba1 levels in microglial cells thereby decreasing the activation of these cells after H/R injury (Figure 3).

Further, we were interested to understand the pathway through which propofol rescued cells from I/R injury. And interestingly, our results indicated that propofol rescued the cells from I/R injury through the downregulation of Cx43. Our results were further strengthened through silencing experiments, which showed that silencing of Cx43 significantly decreased negative effects of H/R injury in both microglial cells and neurons (Figure 4). This evidence further strengthened our understanding of the interactions between neuronal and glial populations along with clarifying the roles of Cx43 in I/R injury. Finally, we could also prove the effect of propofol in rescuing from I/R injury using MCAO models. Evidentially, we could clearly establish that propofol could rescue the MCAO rats by decreasing the infract volume through the decrease of Cx43, Cav3.2, and Iba1 levels (Figure 5). Further, using overexpression models, we could establish that Cx43-OE worsened the injury associated with I/R and propofol treatment could still slightly decrease the cell death and microglial activation through regulation of Cx43 expression. Interestingly previous studies have shown that propofol, an agonist of GABA_A_ receptor and ketamine, an antagonist of glutamatergic channels could efficiently inhibit Cx43 based gap junction and hemichannel based communication. In this study, we further wanted to elucidate its role and understand its association with Cx43. Importantly, all the previous studies have been performed on hippocampal slices or organotypic cultures (Liu et al., 2016), this is the first study to show in an *in vitro and in vivo* MCAO based model, that indeed H/R injury affects neuronal populations through activation of microglial Cx43 and indeed propofol could be used to improve the neuronal morphology and Map2 expression during H/R injury. Therefore, the current study provides insight into the potential use of propofol to treat or prevent cerebral I/R injury.

Our results indicated in Figure 1A, there was almost 40% cell death at 12 h after H/R as well as in Figure 2B only 20% cell death was observed. MTT assayed the viability of living cells (Figure 1A). Cell death includes active cell death—programmed death, apoptosis and passive cell death (cell necrosis). TUNEL-positive cells are apoptosis cells (Figure 2B). Therefore, the apoptosis cells may be less to death cells. Moreover, the TUNEL-positive cells were analyzed according to the photographs, among which there may be a difference. The effect of propofol on H/R induced brain injury may be at least in part due to downregulation Cx43 phosphorylation levels in our study although propofol also shows effect in connexin silenced cells. We would perform further study to explore the exact act of propofol in Cx43 regulation during H/R induced brain injury.

## Data Availability Statement

The data in this study can be obtained from the corresponding author.

## Ethics Statement

The animal study was reviewed and approved by Institutional animal care use and Ethics Committee at the Shanghai Tenth People’s Hospital.

## Author Contributions

TZ, YW, and QX designed and completed experiments and completed the manuscript. TZ, ZT, and JS were responsible for the isolation of primary cells. QJ and PC statistical analyzed the data. XZ directed the study and revisesed the manuscript. All authors contributed to the article and approved the submitted version.

## Conflict of Interest

The authors declare that the research was conducted in the absence of any commercial or financial relationships that could be construed as a potential conflict of interest.
